# Does physical activity counselling enhance the effects of a pedometer-based intervention over the long-term: 12-month findings from the Walking for Wellbeing in the west study

**DOI:** 10.1186/1471-2458-12-206

**Published:** 2012-03-19

**Authors:** Claire F Fitzsimons, Graham Baker, Stuart R Gray, Myra A Nimmo, Nanette Mutrie

**Affiliations:** 1 School of Psychological Sciences and Health, University of Strathclyde, 76 Southbrae Drive, Glasgow G13 1PP, Scotland, UK; 2 Institute of Medical Sciences, University of Aberdeen, Aberdeen, Scotland, UK; 3 School of Sport, Exercise and Health Sciences, Loughborough University, Loughborough, UK

## Abstract

**Background:**

Pedometers provide a simple, cost effective means of motivating individuals to increase walking yet few studies have considered if short term changes in walking behaviour can be maintained in the long-term. The role of physical activity consultations in such interventions is unclear. The purpose of this study was to assess the sustainability of pedometer-based interventions and empirically examine the role of physical activity consultations using long-term results of a community-based walking study.

**Methods:**

79 low active Scottish men and women (63 women and 16 men) from the Walking for Wellbeing in the West intervention study were randomly assigned to receive either: Group 1; pedometer-based walking programme plus physical activity consultations or Group 2; pedometer-based walking programme and minimal advice. Step counts (Omron HJ-109E Step-O-Meter pedometer), 7 day recall of physical activity (IPAQ long), mood (PANAS) and quality of life (EuroQol EQ-5D) were assessed pre-intervention and 12, 24 and 48 weeks after receiving the intervention. Body mass, body mass index and waist and hip circumference were assessed pre-intervention and 12 and 24 weeks after receiving the intervention. Analyses were performed on an intention to treat basis (baseline value carried forward for missing data) using mixed-factorial ANOVAs and follow-up t-tests.

**Results:**

A significant main effect of time (*p *< 0.001) was found for step-counts attributable to significant increases in steps/day between: pre-intervention (*M *= 6941, *SD *= 3047) and 12 weeks (*M *= 9327, *SD *= 4136), *t*(78) = - 6.52, *p *< 0.001, *d *= 0.66; pre-intervention and 24 weeks (*M *= 8804, *SD *= 4145), *t*(78) = - 4.82, *p *< 0.001, *d *= 0.52; and pre-intervention and 48 weeks (*M *= 8450, *SD *= 3855), *t*(78) = - 4.15, *p *< 0.001, *d *= 0.44. Significant effects were found for several variables of self-reported physical activity, mood and quality of life and are discussed. No other significant effects in health related outcomes were found.

**Conclusion:**

Both interventions successfully increased and maintained step counts over 12 months. Physical activity consultations may encourage individuals to be active in other ways beyond walking and to reduce sitting time.

**Trial Registration Number:**

Current Controlled Trials Ltd ISRCTN88907382

## Background

The relationship between an active lifestyle and improved health status is well established, with active individuals enjoying a plethora of health benefits [1]. Thirty minutes of moderate intensity activity on at least five days of the week has been shown to be sufficient to elicit health benefit [2,3]. Current data suggests that less than a third of the adult population in Europe achieve this level of activity [4] and 10.4% of all premature deaths in Europe could be prevented if everyone who is currently inactive became active [5].

Walking interventions can be effective in reducing body weight, body mass index (BMI) and waist and hip circumference [6-8] and may be effective in improving mood, affect [7,9,10] and quality of life [11]. Conversely, some studies have demonstrated that a walking intervention is not sufficient to influence any of these health-related outcomes [12,13]. The reasons for such equivocal results are unclear, therefore determining the potential health benefits that can be achieved through walking is crucial to the public health message.

Pedometers provide a simple, cost effective means of motivating individuals to increase walking [14]. Recent reviews have concluded that pedometer use is associated with an increase in physical activity of approximately 2,000 - 2,500 steps/day and decreases in BMI and body mass [15-17]. Having a step goal has been identified as a key predictor of an increase in activity, although evidence is lacking on the most appropriate goal to use.

Much of the evidence accumulated to date on the use of pedometers is from US based studies with relatively small sample sizes, and predominantly with clinical populations. Additionally, Bravata et al., acknowledged that previous pedometer interventions have incorporated multiple components (e.g. pedometers, step goals, physical activity counselling) and demonstrated heterogeneity in the intensity of the provision of cognitive and behavioural strategies [16]. In order to determine the most effective components the authors recommend empirically examining pedometer use with versus without physical activity counselling. Importantly, the majority of previous studies have been short-term in nature (1 - 15 weeks) [15,16] and evidence is urgently needed to demonstrate if pedometer use is associated with longer term changes in physical activity behaviour and health outcomes [18]. Prior studies have thus far demonstrated mixed effectiveness of pedometer use over a 12-month period [15,16,19-21].

### Walking for Well-Being in the West

The Walking for Wellbeing in the West (WWW) study is a multi-disciplinary community based walking intervention set in the West of Glasgow, Scotland. It was guided by the MRC framework for the evaluation of complex interventions [22] and incorporated behavioural, psychological, physiological, environmental, economic and qualitative elements [23,24]. The study rationale and methods have been described in detail elsewhere [23]. Briefly, WWW was designed to examine pedometer use in low-active adults utilising two approaches; one incorporating additional cognitive and behavioural support through physical activity consultations and one without. Controlled outcome evaluation of the short-term (12 week) findings showed that a pedometer-based intervention combined with a physical activity consultation led to an increase of 3,175 steps/day compared with no significant change in a waiting-list control group [25]. Significant increases in positive affect, subjectively reported walking and decreases in subjectively reported sitting time were reported in the intervention group although no significant changes in anthropometric measures or inflammatory markers of health were found over the short-term [25,26]

The purpose of this paper is to present a comparison of the effects of the two approaches over the longer-term (12 months) on physical activity levels and health outcomes. Thus, we aim to assess the sustainability of pedometer-based interventions and also empirically examine the role of physical activity counselling.

## Methods

### Design of the study

Recruitment for the trial involved leaflets delivered to individual households, posters and flyers displayed in the local area, community stands and advertisements in the local press. Participants were eligible to enter the trial if they were aged 18-65 years, able to understand the rationale behind the trial, were able to walk independently for 5-10 minutes, spoke English, and were in the precontemplation, contemplation or preparation stages of the Transtheoretical model of behaviour change [27] (with respect to meeting the current physical activity recommendations) using an adapted state of change algorithm. All participants were screened using the Physical Activity Readiness Questionnaire (PAR-Q) [28].

### The setting

Interviews, physical activity consultations, completion of questionnaires and data collection from pedometers took place in a specially allocated study room within a University building.

### Participants

**Figure 1:**
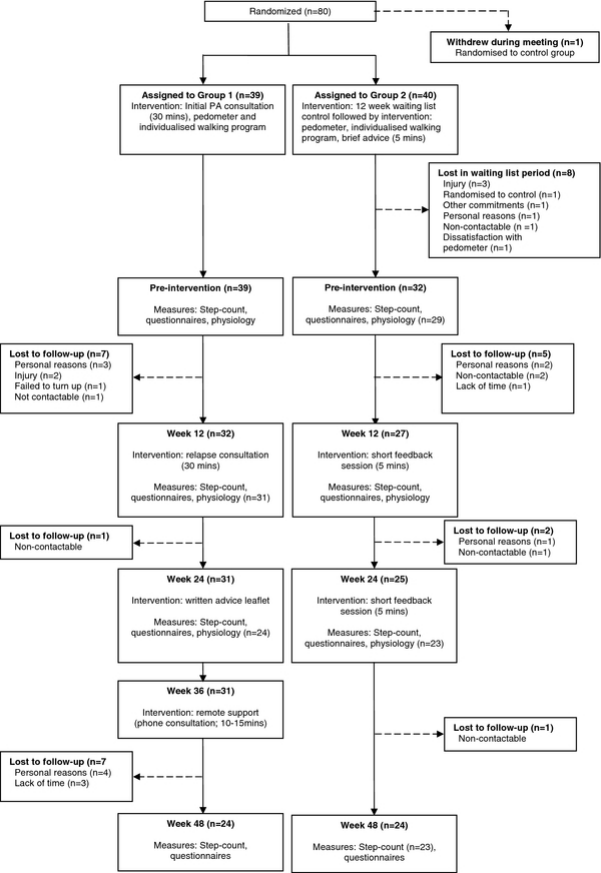
**Participant flow through the study**.

Participant flow through the study is displayed in Figure 1. Seventy-nine individuals (63 females, mean age 49 ± 9 years) were randomised into Group 1; 12-month intervention (n = 39; 31 females) or Group 2; waiting list control for 12-weeks followed by 12-month intervention (n = 40; 32 females). All procedures were approved by University of Strathclyde Ethics Committee (UEC0506/56) and were carried out in accordance with the Declaration of Helsinki. Informed consent was obtained from all participants prior to randomisation.

### Assessment Procedures

The behavioural impact of the intervention was assessed over a 12 month period; therefore the baseline assessment in Group 2 participants was excluded from analysis for the purposes of providing a comparative dataset (baseline data used in circumstances of cases of missing data - see data treatment section). Walking behaviour was assessed using two methods. The primary outcome measure was pedometer step counts (Omron HJ-109E Step-O-Meter). Pedometer data were collected over a 7-day period utilising the HJ-109's memory function. Pre-intervention assessment of step counts was performed using a sealed pedometer to minimise potential reactivity [29]. A secondary measure of physical activity was conducted using the International Physical Activity Questionnaire (IPAQ; long version, self-report) [30]; a 7-day recall utilised to assess the domain and activity type of potential changes in activity, record changes not measured by the pedometer (e.g. swimming) as well as to provide a measure of sitting time.

Affect (an individual's feelings and emotions) was assessed using the Positive and Negative Affect Schedule (PANAS) [31] and quality of life was measured using the Euroqol EQ-5D instrument which incorporates the EQ-5D descriptive system and the EQ VAS [32]. Further details on consistency and reliability of the questionnaires used are available in an earlier trial publication [25]. Body mass was measured on a precision balance (Sartorius, AG Gottingen, accuracy ± 0.001 kg). From these measurements BMI was calculated as height(m)/weight(kg)^2^; height was measured using a standard laboratory stadiometer. Waist-to-hip ratio was calculated from measurements made using a SECA 200 (SECA, Birmingham, UK) measuring tape.

In both groups, walking behaviour, affect and quality of life were recorded at a pre-intervention assessment and subsequently 12, 24 and 48 weeks (considered as 12-months) after receiving the intervention. Anthropometric assessments were taken at pre-intervention, 12 and 24 weeks. Study data were entered in a customised Microsoft Excel database and stored on a secure network drive. Good research practice guidelines were followed for data entry and security [33].

### WWW Intervention

Full details of the WWW intervention, including theoretical framework, physical activity consultation and walking programme, have previously been published [23,25] and an intervention manual is available online http://www.sparcoll.org.uk. A brief summary is provided below. The Transtheoretical Model of behaviour change [27] was used as a theoretical framework for the consultations which followed recommended guidelines [34]. The main cognitive elements of the consultation process focused on goal setting, self monitoring, discussion of barriers, formation of goals incorporating the walking programme and pedometer, enhancing self efficacy, finding social support and relapse prevention/support. A 12 week graduated walking programme aimed to increase participants average daily step count by 3,000 steps/day above baseline on at least five days of the week by week 6, followed by maintenance or subsequent increases if so desired by the participant. The 3,000 steps value is based on the assumption that an adult walking at a moderate pace takes approximately 100 steps/minute (1,000 steps/10 minutes) [35]. An increase of 3,000 steps/day would correspond to an increase of approximately 30 minutes of moderate physical activity, i.e. the physical activity recommendation for adults.

Participants in Group 1 received a 30 minute physical activity consultation at baseline with a trained member of the research team. Following the 12 week walking programme, participants received a second individual physical activity consultation focusing on relapse prevention strategies, encouragement and maintenance of activity. At 24 weeks participants received a written physical activity advice leaflet and at 36 weeks remote support in the form of a short telephone consultation. Participants randomised to Group 2 were allocated to a 12 week waiting list and were requested not to amend their current physical activity levels to act as a true control group. After this time Group 2 received an individualised 12 week walking programme identical to Group 1, five minutes of brief advice and a pedometer but did not receive a physical activity consultation (i.e. the waiting list control group then became a minimal intervention group). The main cognitive elements of the brief advice were goal setting and self monitoring. Immediately following the 12 week walking programme, and also at 24 weeks after receiving the intervention, participants received a short (approximately five minute) feedback session relating to their current levels of walking and use of the pedometer. No further support was provided to this group (see Figure 1).

### Data treatment and statistical analysis

Analysis of the behavioural, psychological and health outcomes was conducted on an intention to treat basis, including both compliers and non-compliers to the intervention using SPSS version 19. Four options were considered when dealing with missing values (baseline values carried forward for missing data; complete case analysis; missing data replaced with average of other group at that time point; missing data replaced with the average of the minimal intervention group (Group 2) at that time point). The results were the same across all options for all outcome measures with the exception of BMI, hip circumference and waist-hip ratio where the following results were found: significant main effect for BMI when missing data replaced with average of other group at that time point; significant main and interaction effects for hip circumference when missing data replaced with average of other group at that time point or when missing data replaced with the average of the minimal intervention group (Group 2); significant main and interaction effects for waist/hip ratio when missing data replaced with average of other group at that time point.

After careful consideration we concluded that the effects found for these variables were small and not clinically meaningful. Therefore, results are presented as baseline value carried forward for missing values. It is our assumption that when people left the trial it is unlikely their activity levels increased any further and more likely that they would return to baseline values. Physical activity is our main outcome measure and there was no change in results for this variable across all different imputation options and a consistent approach to deal with missing data was considered preferable. In addition this is the most conservative form of analysis and reduces our risk of making a Type 1 error.

The primary outcome measure of steps and secondary outcome measures of mood, quality of life and health outcomes were analysed with 4(time) by 2(group) mixed-factorial Analysis of Variance (ANOVA). Significant interaction effects were explored with post-hoc independent t-tests to examine differences between groups in the mean change between time-points (pre-intervention - week 12, week 12 - week 24, week 24 - week 48). Where there was no significant interaction effect, then significant main effects were explored with post-hoc follow-up paired-samples t-tests to examine changes over time in comparison to pre-intervention levels (pre-intervention-week 12, pre-intervention-week 24, pre-intervention-week 48). Data from the International Physical Activity Questionnaire (IPAQ) were transformed (square root) prior to entry into the ANOVA given the non-parametric (positively skewed) nature of the raw data. All *p *values are reported without correction for multiple comparisons: when making multiple comparisons we have exercised caution with interpretation. Data are presented as Mean (M) ± Standard Deviation (SD) unless otherwise stated. A *p*-value of 0.05 was considered as statistically significant with borderline values investigated with caution. Data in tables include descriptive statistics and the main and interaction effect *F*-values from the ANOVA. Significant F-values are highlighted in the tables and post-hoc follow-up test results (F and t values, significance levels, and estimates of effect size - Cohen's *d*) are presented in the text.

## Results

Figure 1 displays the number of participants available at each assessment point. In summary, 79 participants were randomised with 71 participants receiving an intervention. Forty-eight participants completed the final assessment point. Table 1 provides selected baseline characteristics for participants. Preliminary analysis found no significant relationship between age and steps, and gender and steps at any time-point therefore these variables are not included in further analysis.

**Table 1:** Selected baseline characteristics of participants involved in the WWW study

Characteristic	Group 1	Group 2	Whole sample
Number, *n *(%)	39 (49)	40 (51)	79 (100)
Gender (M/F),% (*n*)	21 (8)/79 (31)	20 (8)/80 (32)	20 (16)/80 (63)
Age (years), Mean (± SD)	47.3 (9.3)	51.2 (7.9) ^a^	49.2 (8.8)
Completed University or further education,% (*n*)	56 (22)	83 (33) ^b^	70 (55)
Ethnicity (% White Scottish),% (*n*)	95 (37) ^c^	88 (35) ^d^	91 (72)
SIMD ^e ^(% in top 15%^c^),% (*n*)	13 (5)	8 (3)	10 (8)
Steps, Mean (± SD)	6802 (3212)	7078 (2911)	6941 (3047)

^a ^Independent samples *t*-test indicates significant different between groups: *t*(77) = -1.99, *p *= 0.05

^b ^Pearson's chi-square identifies significant difference between groups: χ ^2 ^(1) = 6.36, *p *= .012

^c ^Other ethnicities, ethnicity (*n*): White Other (1), Caribbean (1)

^d ^Other ethnicities, ethnicity (*n*): Mixed (1), White Irish (1), Pakistani (1), Indian (1), Caribbean (1)

^e ^SIMD (Scottish Index of Multiple Deprivation) top 15% indicates most deprived

### Step counts

**Figure 2:**
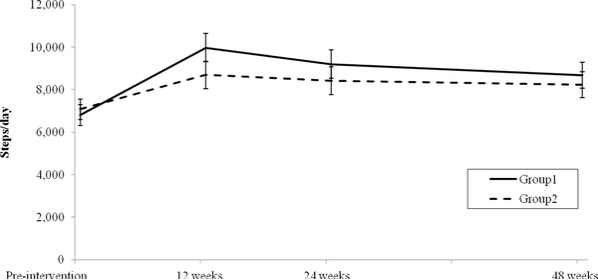
**Step counts (estimated marginal means) for Groups 1 and 2 pre-intervention and at 12, 24 and 48 weeks (error bars represent standard error)**.

Descriptive statistics and ANOVA results for the step count data are presented in Table 2 and Figure 2.

**Table 2:** Mean (SD) for step counts, mood (PANAS + ve, PANAS-ve) and quality of life (EQ-5D, EQ-VAS)

	N	Group	Mean (SD)				RM ANOVA F-value	
			Pre- intervention	Week 12	Week 24	Week 48	Time	Interaction
**Steps**	79	1	6802 (3212)	9977 (4669)	9201 (4468)	8678 (3871)	17.25**	1.75
		2	7078 (2911)	8693 (3483)	8417 (3821)	8228 (3874)		
**PANAS + ve**	79	1	31.2 (6.7)	33.5 (7.4)	32.7 (7.5)	33.9 (8.3)	4.37*	3.51*
		2	31.3 (7.6)	32.1 (6.8)	34.7 (7.5)	31.7 (6.4)		
**PANAS -ve**	79	1	20.1 (7.2)	19.1 (7.0)	19.8 (8.2)	20.5 (7.6)	0.09	1.51
		2	18.8 (7.5)	19.5 (7.5)	18.4 (7.3)	18.2 (8.1)		
**EQ-5D**	79	1	0.88 (0.12)	0.89 (0.12)	0.87 (0.16)	0.89 (0.12)	0.15	0.64
		2	0.88 (0.12)	0.88 (0.17)	0.88 (0.12)	0.87 (0.17)		
**EQ-VAS**	79	1	65.4 (18.3)	69.5 (17.8)	73.3 (18.2)	71.1 (19.7)	4.01*	1.19
		2	70.7 (18.6)	72.7 (16.3)	73.2 (18.1)	72.2 (17.6)		

* indicates significance at *p *< 0.05

** indicates significance at *p *< 0.001

A significant main effect of time (*p *< 0.001) was found for step-counts but there was no significant interaction effect (see Table 2). Post-hoc tests showed significant increases in steps/day between: pre-intervention (*M *= 6941, *SD *= 3047) and 12 weeks (*M *= 9327, *SD *= 4136), *t*(78) = - 6.52, *p *< 0.001, *d *= 0.66; pre-intervention and 24 weeks (*M *= 8804, *SD *= 4145), *t*(78) = - 4.82, *p *< 0.001, *d *= 0.52; and pre-intervention and 48 weeks (*M *= 8450, *SD *= 3855), *t*(78) = - 4.15, *p *< 0.001, *d *= 0.44. There was no significant difference between groups in the number of participants who achieved a weekly step increase of ≥ 15,000 steps 12 months after receiving an intervention (Group 1 13/39 (33%); Group 2 11/40 (28%); *χ*^2 ^= 0.189, *p *= 0.664).

### International Physical Activity Questionnaire (IPAQ)

**Figure 3:**
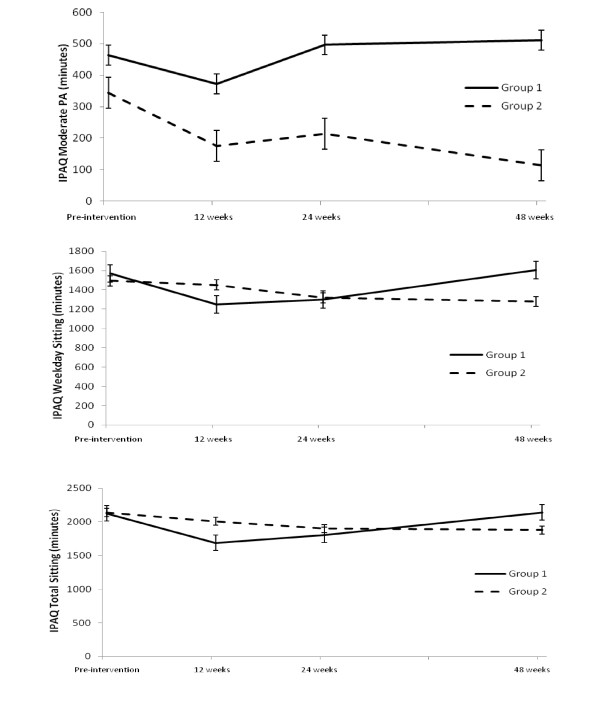
**Self reported (IPAQ) total moderate physical activity, weekday and total sitting for Groups 1 and 2 (means are model-predicted values, error bars are standard error) pre-intervention and at 12, 24 and 48 weeks**.

Descriptive statistics and ANOVA results for the IPAQ are presented in Table 3 and Figure 3.

**Table 3:** Descriptive statistics for IPAQ variables (minutes), median (and range).

		Time Point				RM ANOVA F value	
	Group	Pre-int	Week 12	Week 24	Week 48	Time	Interaction
Work related physical activity							
Walking	1	0 (1620)	0 (2520)	0 (1680)	0 (840)	0.74	2.43
	2	0 (1650)	0 (1200)	0 (1350)	0 (1800)		
Moderate PA	1	0 (1500)	0 (900)	0 (1680)	0 (900)	1.38	0.02
	2	0 (600)	0 (1500)	0 (1500)	0 (1500)		
Vigorous PA	1	0 (1080)	0 (1800)	0 (1680)	0 (1080)	0.93	0.97
	2	0 (540)	0 (480)	0 (480)	0 (150)		
Total	1	0 (3000)	30 (4680)	20 (4320)	0 (2520)	1.41	1.38
	2	0 (2730)	0 (2580)	0 (2550)	0 (2550)		
Transport physical activity							
Walking	1	105 (1680)	140 (900)	150 (720)	150 (1680)	1.56	0.55
	2	70 (1680)	103 (1680)	95 (1680)	80 (1680)		
Cycling	1	0 (0)	0 (0)	0 (0)	0 (0)	1.56	1.56
	2	0 (40)	0 (60)	0 (60)	0 (40)		
Total	1	105 (1680)	140 (900)	150 (720)	150 (1680)	1.56	0.59
	2	70 (1720)	103 (1720)	95 (1720)	80 (1720)		
Housework physical activity							
Moderate inside home	1	210 (2100)	150 (840)	360 (900)	240 (2100)	3.76*	2.38
	2	120 (1260)	0 (840)	20 (1500)	0 (840)		
Moderate outside home	1	0 (2100)	0 (1680)	30 (1260)	30 (2100)	1.44	2.39
	2	0 (840)	20 (840)	30 (1800)	0 (840)		
Vigorous outside home	1	0 (840)	0 (840)	0 (840)	0 (840)	1.34	0.70
	2	0 (360)	0 (900)	0 (960)	0 (360)		
Total	1	360 (4200)	300 (2520)	400 (2340)	420 (4200)	2.02	1.54
	2	203 (2520)	170 (2520)	270 (2520)	240 (2520)		
Leisure time physical activity							
Walking	1	40 (840)	100 (840)	120 (1260)	90 (2100)	1.77	1.25
	2	16 (840)	60 (420)	30 (600)	55 (840)		
Moderate PA	1	0 (360)	0 (60)	0 (300)	0 (210)	1.69	0.76
	2	0 (180)	0 (60)	0 (90)	0 (240)		
Vigorous PA	1	0 (180)	0 (120)	0 (480)	0 (720)	2.40	1.71
	2	0 (600)	0 (140)	0 (360)	0 (240)		
Total	1	60 (840)	120 (840)	120 (1260)	160 (2610)	0.84	2.12
	2	60 (840)	75 (420)	40 (780)	65 (840)		
Combined domains							
Total walking	1	225 (3360)	290 (2850)	250 (2310)	240 (3360)	2.32	0.39
	2	155 (1925)	235 (1740)	204 (1740)	230 (1845)		
Total moderate PA	1	420 (4380)	405 (2760)	525 (4020)	465 (4380)	2.64	3.02*
	2	263 (2100)	95 (2400)	105 (3300)	8 (1800)		
Total vigorous PA	1	0 (1080)	0 (1800)	0 (1680)	0 (1080)	0.73	2.15
	2	0 (600)	0 (480)	0 (480)	0 (240)		
Total PA	1	690 (6300)	840 (5415)	845 (7800)	870 (6200)	1.08	0.34
	2	578 (4270)	730 (4330)	828 (4330)	570 (4275)		
Time Sitting							
Weekday	1	1500 (3750)	1200 (3900)	1200 (4165)	1500 (3900)	3.37*	4.16*
	2	1500 (2850)	1425 (4050)	1200 (3300)	1200 (3300)		
Weekend	1	480 (1320)	360 (1200)	480 (1320)	480 (1320)	6.66**	0.23
	2	600 (1320)	480 (1320)	480 (1560)	600 (1560)		
Total	1	1980 (4650)	1680 (5100)	1680 (4645)	1830 (4860)	4.60*	3.56*
	2	2100 (3630)	1845 (4170)	1770 (4860)	1770 (4860)		

RM ANOVA performed on transformed data

* indicates significance at p < 0.05

** indicates significance at p < 0.001

There was a significant interaction effect found for total moderate PA (*p *< 0.05). Follow-up independent t-tests found no significant differences between groups in mean change between any time-points.

There was a significant interaction effect found for weekday sitting (*p *< 0.05). Post-hoc tests revealed there was a significant difference between: Group 1 (Mean change = -325.00, SD = 690.47) and Group 2 (Mean change = -36.25, SD = 520.97) for mean change between pre-intervention and week 12, *t*(77) = - 2.14, *p *= 0.035, *d *= 0.48; and between Group 1 (Mean change = 44.62, SD = 193.06) and Group 2 (Mean change = 27.00, SD = 271.05) for the change between week 24 and week 48, *t*(77) = 2.068, *p *= 0.042, *d *= 0.47.

There was a significant interaction effect found for total sitting (*p *< 0.05). Post-hoc tests revealed there was a significant difference between: Group 1 (Mean change = -451.15, SD = 848.22) and Group 2 (Mean change = -130.25, SD = 567.75) for mean change between pre-intervention and week 12, *t*(77) = - 2.03, *p *= 0.046, *d *= 0.46.

A significant main effect of time was found for moderate housework inside the home (*p *< 0.05). Paired t-tests showed a significant decrease in moderate housework inside the home between pre-intervention (*M *= 336.46, *SD *= 423.22) and 12 weeks (*M *= 223.89, *SD *= 264.75), *t*(78) = - 2.94, *p *= 0.004, *d *= 0.34. A significant main effect of time was also found for weekend sitting (*p *< 0.001). Paired t-tests found a significant decrease between pre-intervention (*M *= 615.70, *SD *= 333.22) and 12 weeks (*M *= 505.82, *SD *= 276.58), *t*(78) = - 4.21, *p *= 0.030, *d *= 0.35; and between pre-intervention and 24 weeks (*M *= 555.19, *SD *= 325.60), t(78) = - 2.22, *p *= 0.030, *d *= 0.19.

### Mood: Positive and Negative Affect Schedule (PANAS)

**Figure 4:**
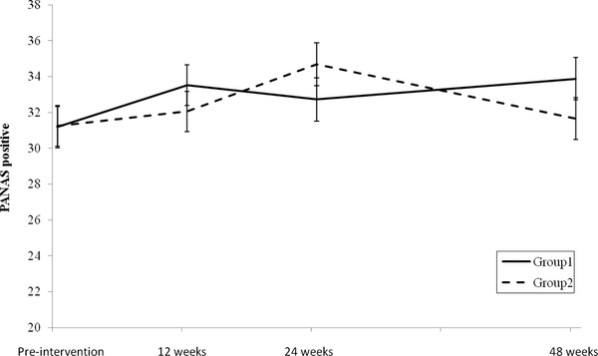
**PANAS positive (estimated marginal means) for Groups 1 and 2 pre-intervention and at 12, 24 and 48 weeks (error bars represent standard error)**.

Descriptive statistics and ANOVA results for the PANAS are presented in Table 2 and Figure 4.

A significant interaction effect and a significant main effect were found for PANAS positive (*p *< 0.05) (Table 2). Follow-up independent t-tests found no significant differences between groups in mean change between any time-points.

### Quality of Life: EQ-5D and EQ-VAS

Descriptive statistics and ANOVA results for the EQ-5D and EQ-VAS are shown in Table 2. A significant main effect of time was found for the EQ-VAS sub-scale (*p *< 0.05). Paired t-tests found significant increases between pre-intervention (*M *= 68.1, *SD *= 18.5) and 24 weeks (*M *= 73.2, *SD *= 18.0), *t*(78) = - 3.152, *p *= 0.002, *d *= 0.28 and pre-intervention and 48 weeks (*M *= 71.6, *SD *= 18.5), *t*(78) = - 2.601, *p *= 0.011, *d *= 0.19.

### Anthropometric measures

Descriptive statistics and ANOVA results for the anthropometric measures are presented in Table 4. There were no main or interaction effects found for body mass, BMI, waist circumference, hip circumference or the waist: hip ratio.

**Table 4:** Mean (SD) and RM ANOVA for health related outcomes

	Group 1			Group 2			RM ANOVA F-value	
	Pre-intervention	Week 12	Week 24	Pre-intervention	Week 12	Week 24	Time	Interaction
Body mass (kg)	78.86 (15.58)	79.12 (15.24)	79.33 (15.40)	79.53 (17.16)	79.30 (17.37)	79.57 (17.18)	0.82	0.73
BMI (kg/m^2^)	28.54 (4.83)	28.64 (4.79)	28.72 (4.85)	29.47 (6.19)	29.37 (5.97)	29.48 (5.93)	0.89	0.90
Waist Circumference (cm)	89.48 (12.64)	89.79 (12.70)	90.46 (13.03)	90.91 (15.58)	90.19 (15.00)	90.21 (14.01)	0.36	2.16
Hip Circumference (cm)	108.89 (8.77)	108.55 (9.70)	108.86 (9.73)	110.20 (11.77)	109.79 (11.37)	109.21 (11.23)	1.62	1.65
Waist:Hip Ratio	0.82 (0.08)	0.83 (0.08)	0.83 (0.08)	0.82 (0.09)	0.82 (0.09)	0.83 (0.09)	1.88	0.82

## Discussion

The main aim of this study was to examine the effects of two approaches to delivering a pedometer-based intervention, one including physical activity consultations and one without, on physical activity levels in low active Scottish men and women. The results show that short-term increases in physical activity, typically observed in pedometer-interventions, [16] can be maintained over the longer-term. Both intervention approaches utilised in this study led to an increase in step-counts that was maintained over 12-months; collectively an increase of 1,509 steps/day was observed representing approximately an additional 15 minutes of walking/day (or 105 minutes walking/week).

This increase in physical activity levels of 22% above pre-intervention values compares favourably with recent systematic review findings where the overall increase for studies, typically short-term in nature, was 26.9% [16]. Previous studies have shown mixed evidence of the utility of the pedometer over the longer-term. This study of a community population confirms the findings of previous studies involving clinical samples where short-term increases are maintained over the longer-term [21,36]. Other studies involving workplace samples have demonstrated complete regression to baseline values although the shorter initial intervention (4-weeks) of these studies may explain the conflicting findings with those found here [19,20].

The synthesis of the literature on the effectiveness of pedometers conducted by Bravata et al., found that physical activity counselling in conjunction with pedometer use did not increase steps walked per day [16]. The findings of the current study suggest that exposure to physical activity consultations in the intervention provided a modest advantage compared to those who did not receive these. For example, both the percentage increase from pre-intervention levels to 12-months (28% versus 16%) and the percentage meeting steps-count goals at 12 months (33% versus 28%) were higher in Group 1 participants although not statistically significant. This is also evident in the maintenance of self-reported total moderate PA in Group 1 in comparison to the decrease found in Group 2 over time. Qualitative findings from this study also support the benefits of the consultations in providing support and encouragement and participants expressing concern about sustaining levels of walking once this support came to an end [24].

Sedentary behaviour is an increasingly researched area although few interventions exist that specifically target this activity in adults [37]. Although not an aim of this study, participants in Group 1 self-reported greater reductions in the short term in time spent sitting than Group 2. A reduction in sitting time is consistent with previous pedometer-based interventions [13,38]. This provides further evidence to the suggestion that the physical activity consultations provided additional benefit to participants.

No significant changes were observed in any of the anthropometric measures. There are several possibilities as to why positive effects were not observed in our participants. On average participants' values for measured outcomes were within the normal healthy range at pre-intervention assessment. Changes in these measures, therefore, could not be expected, compared to changes that might be expected in obese or other clinical populations. Furthermore it has been suggested that to elicit weight loss, between 60-90 minutes/day of moderate intensity exercise is required [38] which equates to in excess of the 3,000 steps/day goal of this study. Intensity is also an important factor in determining the health benefits of exercise. It is possible that the intensity of the physical activity increases observed in this study was not at sufficient intensity to stimulate health benefits. We recognise a limitation of this study is the lack of anthropometric data at 48 weeks. Due to a member of staff leaving we had insufficient capacity within the research team to conduct all the anthropometric assessments at the 48 week follow up. We were unable to appoint and train a replacement within the time scale available. It is possible that anthropometric variables may have changed over a longer time scale. Overall the findings of this study and similar community based studies [7,8,13] are inconclusive with respect to beneficial changes in health outcomes following successful behaviour change.

However, both interventions reported improvements in affect via PANAS positive scores and self-related health as measured by the EQ-VAS. This provides support for walking as an activity that improves people's mood and well-being. Such positive affect may be linked to intrinsic motivation, thus potentially enhancing adherence [39].

A significant challenge with a longitudinal study of this nature is to maintain participant numbers throughout the intervention and minimise drop out. At final assessment in this study, 48 of 79 participants (61%) returned. It is difficult to find comparable studies in the literature against which to compare retention rates over a similar study duration. Sugiura et al., evaluated the effects of a 24 month intervention in menopausal women [36]. Of 48 participants originally randomised to an intervention condition, 27 were retained to 24 months representing a retention rate of 56%. Although our retention rate at 12 months compares favourably with Sugiura et al., incentives for continued participation, which were not utilised in the WWW study, may have helped to reduce drop-outs.

### Study strengths and limitations

This is the first pedometer study to track participants walking levels over 12 months in response to two interventions, thus allowing for an empirical investigation into potential additional effects of physical activity consultations. This study is also one of the first to provide follow-up measurements to 12 months in a community sample thus investigating the issue of maintenance in pedometer-based interventions. We are currently exploring implementation and translation of this intervention into other settings and also conducting on-going follow ups for WWW at 24 and 36 months. This study therefore provides an important contribution to the area of public health as it provides evidence of low cost and minimal contact interventions (through the form of a pedometer and a walking programme) having the capacity to produce behaviour change that is maintained over the long-term [24].

We chose to analyse the results on an intention to treat basis. This is the most conservative estimate of missing values. Analysis based upon those who successfully completed a walking intervention, rather than an 'intention to treat' approach, has previously been reported as a weakness in the literature and reduces the degree to which findings can be applied to a population setting.

To permit a direct comparison between both interventions we chose not to have a control arm throughout the study. We questioned how ethical and practical it would be to ask someone who had volunteered for a walking study and wished to increase their physical activity levels to remain on a waiting list control condition for 12 months. We recognise however, the lack of a control condition throughout is a limitation of the study. We did however, utilise the minimal intervention group as a waiting list control group for the first 12 weeks; during this time no significant change in physical activity levels occurred [25].

Despite initial attempts to engage a deprived population, [23] the participants in this study were white, well educated, middle aged and predominantly female which is consistent with previous studies [15,16]. It is therefore possible that the observed effects may be different in other populations. We chose not to stratify our analysis by gender given the low number of males but we acknowledge that future research should address this issue. A significantly higher proportion of participants in Group 2 were educated to University or further education level. It could be hypothesised that this higher level of education contributes in some way to the increase in steps found in Group 2 despite a more minimal intervention. However, we have analysed our results according to education level and found no evidence that education level was associated with the level of change in step-counts. Group 2 participants are also significantly older by approximately 4 years. We do not consider this difference to be clinically meaningful in terms of our outcome measures, and exploratory analysis found no evidence that age was associated with step-counts at any-time point.

## Conclusion

In summary, this study has demonstrated that it is possible to increase and maintain walking levels in low active Scottish men and women over 12 months using pedometer-based interventions. The addition of a physical activity consultation focused on walking seems to have had limited additional benefit in relation to step counts or health indices but the consultation may have encouraged individuals to be active in other ways beyond walking and to reduce sitting time.

## Abbreviations

ANOVA: Analysis of Variance; BMI: Body mass index; IPAQ: International Physical Activity Questionnaire; M: Mean; Mdn, Median; MRC: Medical Research Council; PANAS: Positive and Negative Affect Schedule; PAR-Q: Physical Activity Readiness Questionnaire; R: Range; SPARColl: Scottish Physical Activity Research Collaboration; SD: Standard deviation; WWW: Walking for Well-being in the West.

## Competing interests

The authors declare that they have no competing interests.

## Authors' contributions

CF drafted the manuscript, participated in the coordination of the study, assisted with collection of the health related outcome data and performed statistical analysis of step counts, IPAQ, mood and quality of life measures. GB was responsible for data collection, performed the physical activity consultations, assisted with statistical analysis and drafting of the manuscript. SG was responsible for collection and analysis of health related outcome data and drafted the health related outcome sections of the manuscript. MN led the health related component of the study and contributed to data collection and analysis. NM (on behalf of SPARColl) as principal investigator conceived and managed all elements of the study. All authors contributed to the design of the study and interpretation of emerging findings. All authors provided feedback during the drafting of the manuscript and read and approved the final version.

## Pre-publication history

The pre-publication history for this paper can be accessed here:

http://www.biomedcentral.com/1471-2458/12/206/prepub
